# Folsom Transplant Blues: What is Wrong with Offering the Incarcerated Shorter Sentences for Donating Organs and Bone Marrow?

**DOI:** 10.1017/jme.2025.44

**Published:** 2025

**Authors:** Andreas Albertsen, Jens Damgaard Thaysen

**Affiliations:** 1 Political Science, Aarhus Universitet, Aarhus, Denmark; 2 CEPDISC. The Centre for the Experimental-Philosophical Study of Discrimination, Aarhus Universitet, Aarhus, Central Denmark Region, Denmark; 3 Department of Culture and Learning, Aalborg Universitet, Aalborg, North Denmark Region, Denmark

**Keywords:** organ donation, organ markets, living donation, punishment, Incarcerated organ donors

## Abstract

In Massachusetts, a proposed bill would reduce the sentence of those incarcerated who become living donors of either organs or bone marrow. We outline two concerns with such a proposal, which relate directly to the content of the proposal (as opposed to broader debates about payment for organs and validity of consent obtained from the incarcerated). The first of these concerns is about equality of opportunity. The proposal provides the opportunity for a sentence reduction for some but not for others – and the distribution of these opportunities reflects circumstances largely beyond the control of the incarcerated. The second concern is that the proposal may conflict with why we punish in the first place. The proposal is at odds with the non-consequentialist general deterrence defended by Tadros, retributivism, and communicative theories of punishment. Among the theories examined, only the purely consequentialist version of general deterrence might find the practice palatable. The upshot of the latter observation is that the proposal presupposes the truth of a purely consequentialist theory of punishment and sets aside others.

## Introduction

In February 2023, Bill H.2333 was submitted to the State House of Massachusetts. It is entitled *An Act to Establish the Massachusetts Incarcerated Individual Bone Marrow and Organ Donation Program.* ^1^ Such a program would expand organ and bone marrow donation opportunities for the incarcerated. However, the bill came with an additional, controversial element: It would “allow eligible incarcerated individuals to gain not less than 60 and not more than 365 day reduction in the length of their committed sentence … on the condition that the incarcerated individual has donated bone marrow or organ(s)”.^2^ A committee would decide the length of this reduction.

The logistics of testing, matching, and eventually undergoing surgery are often challenging for the incarcerated. Establishing an organ donation program within the prison system would help overcome these barriers.^3^ The expansion of donation opportunities is not uncontroversial.^4^ However, offering a shorter sentence to those who donate raised much more controversy.^5^ In Massachusetts, one of the bill’s original supporters has promised to remove this element in light of the criticism.^6^

Since paying for organ donation or introducing organ markets is controversial, it is hardly surprising that this proposal was widely opposed. This article contributes to the ongoing discussion of the proposal by developing and discussing two specific critiques, each of which relates directly to the proposal at hand. Our approach thus departs from other possible approaches to the proposal, which would treat it as a sub-discussion of already existing debates. One alternative would be to see whether some of the general concerns raised in the discussion on payment for organs are applicable here.^7^ Another approach would be to draw on the existing literature on what the incarcerated can consent to. That question has been addressed, for example, in relation to whether the incarcerated should be allowed to participate in paid medical research.^8^ But we do not wish to rehearse these debates in this particular context. We are interested in bringing out concerns related to the proposal instead of discussing overall concerns with paid organ donation or the validity of consent from incarcerated. We suspect that those who assert that the incarcerated can provide valid consent and those who deny it will reach a similar conclusion in this case.^9^ And we envision that this case will not provide new insights into the general question of payment for organ donation.

In contrast, this article suggests that there are unique and specific worries associated with this particular proposal **—** irrespectively of the merits of paid organ donation **—** and in addition to worries one might have about the consent of the incarcerated. These problems pertain to inequality of opportunity and broader perspectives of the morality of punishment. This article is dedicated to the discussion of these problems. The argumentative strategy of the article is not to evaluate and defend a particular theory of equality of opportunity or position regarding the morality of punishment. Instead, the purpose is to examine whether the proposal conflicts with a wide range of understandings of these.

## The merits of the proposal

Before criticizing the proposal, we should pause and consider why the proposal might be worth considering in the first place. The first reason is that the proposal might help procure more organs. If the incarcerated would use the opportunity, the donated organs would prolong and improve the lives of some of those currently waiting for an organ transplant. So, just as for any other proposal that would increase the number of organs available, this should count as an advantage of the proposal. In the US, 100,000 people are currently waiting for an organ transplant.^10^

Of course, the extent to which the system would procure more organs and whether donating would result in those improvements is somewhat speculative. Moreover, at least in principle, these benefits could be realized by setting up the bone marrow and organ donation program *without* offering sentence reduction. However, the added measure of a sentence reduction might increase the number of people willing to become living organ donors or bone marrow donors.

Last, reducing sentences might in itself be beneficial. It could be that a reduction in people’s sentences would make them return as productive members of society and reduce the costs associated with incarceration. Having mentioned some potential benefits of the proposals, we now turn to two kinds of worries.

## Problems from unequal opportunity

Theories of equality of opportunities reflect the notion of fairness — that it is important that we are equally situated in terms of the range of options available to us and that external factors do not unduly influence our ability to seize these options successfully. Following John Roemer, a person has an equal opportunity for achieving some good *x*, if and only if, the extent to which he achieves *x* reflects his chosen effort as opposed to his circumstances.^11^ This broad formulation captures the content of a wide subset of theories of equality of opportunity.^12^

On such understandings of equality of opportunity, the proposal creates problematic inequalities of opportunity for achieving a sentence reduction through conducting either a bone marrow or an organ donation. To see why this is the case, recall that the sentence reduction is conditional on the performance of an actual organ(s) or bone marrow donation. It is not enough to declare one’s willingness to do so. This creates inequality of opportunity because not everyone can donate an organ or bone marrow. People are not equally situated to become donors.

Not all such inequalities in donation opportunities are necessarily problematic. Consider, for example, an incarcerated person who donated a living kidney to a relative before incarceration.^13^ This person would not have the same opportunities as other incarcerated people for receiving a reduced sentence because he has no “extra” kidney to donate.^14^ However, that might not be a problem from the perspective of equality of opportunity, as this person’s worse opportunity in prison reflects previous choices. Assuming that the previous choice was voluntary, this may not constitute an unequal opportunity.

However, we can easily imagine cases of persons who lack the opportunity to donate for reasons that constitute more clear-cut instances of unequal opportunities. After all, some are not considered medically suitable donors because of circumstances outside their control. As an illustration, consider the current regulations at the Transplant Center at Massachusetts General Hospital.^15^ For kidney donations, there is an age limit: 25–75. Those aged 18–25 are only considered eligible if they have a close relationship with the recipient. Such limits remove the option of living organ donation for those outside the age limit, including those under 25 who do not know anyone in need of a transplant who could benefit from their kidney (i.e., where the kidney matches). Other requirements pertain to the health of the potential donor. The body mass index must be 35 or less; a history of major medical conditions, such as heart, lung, and liver diseases, also disqualifies a donor, and so does active cancer, uncontrolled diabetes, and high blood pressure (if not well-controlled).^16^ For living liver donations, “donors must be between 18 and 55 and have no serious medical problems”.^17^ While not explicitly listed in the regulations, a history of heavy substance use may also mean you cannot donate a liver.^18^ These requirements reflect that you need to be in a particular physical condition to become an organ donor and that the quality of the donated organs should also be sufficient to benefit the organ recipient. In addition, conducting a living organ donation also requires some probing into whether the donor would be mentally fit to perform the donation.^19^

One reaction to these inequalities would be to point out that organ donation is not the only route to sentence reduction. After all, the proposal would also establish a bone marrow donation program. However, this reply does not alter the picture. The reason for this is that a similar problem arises here. Living bone marrow donation programs also have requirements for age, illnesses, medical history, and physical condition.^20^ So a familiar pattern emerges.

This leaves us with a situation where there would be a significant inequality of opportunities if the proposal were introduced. Among those incarcerated who would want a sentence reduction, many would not be able to obtain it. Whether because of age, medical condition or, to some degree, past behavior, the described barriers mean that sentence reductions will be a good not available to some because of circumstances outside of their control, rendering them problematic from the perspective of the account of equality of opportunity employed here (and indeed on many such accounts).

How much of an argument against the proposal does the identified problem pose? Some would perhaps suggest that there is no reason to be alarmed here. Providing opportunities for some but not for others is only sometimes a cause for concern. This is true but proves little in this context. To see that it is true, consider a case where donation opportunities are increased by allowing the incarcerated to donate, but where such donations are *not* rewarded by a sentence reduction. In the absence of the reward, few would be troubled by providing the opportunity to donate to some, even if others were not eligible to donate. But when connecting the opportunity to donate with a sentence reduction, things look rather different. The first thing to note is that the reward is substantial. According to a recent statistic from the US Department of Justice, the “average time served by state prisoners released in 2018, from their date of initial admission to their date of initial release, was 2.7 years”.^21^ Reducing sentences by 2 to 12 months is a substantial benefit. One which it is reasonable to assume that most incarcerated would want. But a benefit which is, predictably, only attainable to some under the current proposal.^22^

In our view, the unequal opportunity argument just presented can only be a partial reason against introducing the proposal under discussion. The reason for this is that the problem of inequality of opportunity can, at least in principle, be mitigated in several ways. One important thing to note here is that donating an organ would not be the only route to a sentence reduction. Several such routes are already in place in the US. These include so-called Good Time Credits available for those incarcerated in Federal facilities, where your sentence is shortened if you have a high school diploma or GED (or are studying to get one while incarcerated) and avoid disciplinary problems.^23^ Other examples are community outreach programs known as the Residential Drug Awareness Program (RDAP)^24^ and Compassionate Releases, where an incarcerated can argue that changed circumstances make their continued imprisonment unjust.^25^ The existence of such proposals ensures that those who cannot become organ donors can pursue a sentence reduction through other contributions. And, notably, Compassionate Releases would (if available) be a route open to individuals who are too sick and old to be eligible for donating an organ. It would not help those who are too young to donate or those who are too ill to donate but not ill enough to receive a compassionate release.

In sum, introducing the proposal would introduce a problematic inequality of opportunity for a significant good. This provides a pro tanto reason against the proposal, the strength of which can be mitigated by ensuring that other routes for sentence reduction are also available for those who cannot donate organs or bone marrow. The extent to which such measures can, in fact, do so depends on how they are implemented and whether there are, for example, racial or social disparities in who is able to successfully navigate these alternative options.

## Problems from the morality of punishment

Apart from being concerned with the equality of opportunity associated with offering a sentence reduction, there are also potential problems if we look at the proposal from broader theories of punishment. This section asks whether prominent theories of why we punish are consistent with the proposal.

As outlined in the introduction, our argumentative strategy is to show that a broad subset of relevant theories of punishment are incompatible with offering sentence reductions to those who donate, thereby indicating that several positions within the philosophy of punishment are committed to rejecting Bill H.2333. Thus, we will present theories of punishment and discuss their implications for Bill H.2333 without attempting to evaluate the relative merits of the theories of punishment themselves.

Unfortunately, it is impossible to discuss every variation of every theory of punishment. We discuss standard versions of *general deterrence* (in a consequentialist and non-consequentialist version, respectively), *retributivism*, and *communicative theories of punishment.* These are arguably the most prominent theories of punishment. While we do not discuss *mixed theories of punishment*, such theories are, after all, characterized by mixing the type of theories we do discuss, so our discussion should apply to those as well. Still, we must concede that many theories and variations of theories of punishment are not discussed but we hope that the theories we do discuss are interesting enough for a survey of their implications for Bill H.2333 to be interesting.

### General deterrence

According to general deterrence theories, the primary purpose of punishment is to prevent crime by sustaining the credibility of a threat of punishment that deters potential criminals from offending.^26^ General deterrence is an instrumentalist approach to punishment. Punishment is justified because it brings about something good, namely the prevention of crime, which is good insofar as criminalized conduct is bad. This instrumental justification of punishment is often, but need not be, rooted in more general consequentialism,^27^ according to which punishment is justified because the good brought about by punishment outweighs the bad it brings about.^28^ The main good punishment can bring about is crime prevention.^29^

Evaluating Bill H.2333 from the perspective of consequentialist general deterrence thus involves assessing whether the combined good brought about by adopting the proposal outweighs the bad. Assuming some incarcerated individuals will wish to avail themselves of the proposed program, enacting Bill H.2333 would bring about the good of having more kidneys for transplant, which is also the main consideration its proponents advance in its favor. Furthermore, the 60–365-day reduction in the sentence of those who donate avoids the bad that would have been brought about by incarcerating those persons for the relevant amount of time. The proposal would bring about the bad of diluting the crime-preventing effects of punishment as well as the pain and other adverse consequences suffered by the live donors. There is reason to think that Bill H.2333 would be justified from the perspective of consequentialist general deterrence. First, the deterrent effect of *the length of sentences* (as opposed to the risk of apprehension) may be quite weak.^30^ As such, it is not clear that the possibility of shortening one’s sentence by a year will reduce the deterrent effect of criminal prohibitions, especially if we take into account that the sentence reduction comes at a cost: the loss of a kidney to the incarcerated. Note, however, that unless the prospect of having to give up a kidney in exchange for a sentence reduction contributes significantly to maintaining the deterrent effect of the law, consequentialist general deterrence provides no real reason to insist that the organ donated in exchange for a reduction in the sentence served by X must be donated by X rather than another person willing to donate in exchange for a reduction in X’s sentence (e.g., a spouse or close family member).

As mentioned above, proponents of general deterrence need not be full-fledged consequentialists. Victor Tadros has recently defended an instrumentalist, but non-consequentialist, version of general deterrence. According to Tadros, criminal wrongdoers incur two enforceable duties in virtue of their wrongdoing: a duty to recognize that they have done wrong and a duty to prevent their victims from being harmed by others in the future, which Tadros argues can be extended to a more general duty to prevent others from being harmed by crime in the future.^31^ The fact that criminal wrongdoers incur these duties (particularly the latter) justifies punishing offenders for the sake of general deterrence, even though doing so harms them as a *means* to prevent crime, because offenders have an enforceable duty to serve this end anyway.^32^ On this view, the question becomes whether offenders can discharge the enforceable duties they incur as a result of criminal wrongdoing by donating an organ.

One can (partially) discharge a duty to prevent further harm to victims of crime by donating an organ to a victim of crime in need of a transplant. However, from this perspective, Bill H.2333 ought to earmark any organs donated under the suggested scheme for victims of crime. But it does not. Tadros also points out that while the duties incurred through criminal wrongdoing justify using offenders as a means to protect the victims of crime from further harm, it is not justified to punish offenders “as a means to solve problems unrelated to those they caused.”^33^ Since organ shortage is not driven by criminal offending to any significant extent, the duties to prevent further harm to their victims incurred by criminal wrongdoers cannot be discharged by contributing to a solution of this problem.^34^

Perhaps donating an organ could contribute to discharging a duty to recognize that one has done wrong by serving as an expression of remorse. However, the offer of a sentence reduction will undercut the ability of offers to donate to serve as expressions of remorse, since creating a prudential reason to donate will make it harder to tell whether the offender is motivated by genuine remorse.^35^ It would probably be good to make it easier for the incarcerated to donate. Still, the proposal that we reward donations with sentence reductions made by Bill H.2333 is at odds with the non-consequentialist general deterrence of Tadros.

### Retributivism

According to *retributivism*, the purpose of punishing offenders is retribution in proportion to their moral desert, which is itself a product of the wrongness of the offender’s culpability for committing that offense.^36^ The most straightforward way to justify reducing the sentences of those willing to donate from a retributivist perspective is to argue that removing an organ can be part of giving them what they deserve (by making them suffer in a punishment-appropriate way).^37^ Although a nephrectomy is hardly pleasant and might be viewed as causing suffering, this is not a promising strategy for several reasons. First, retributivists tend to claim that punishment institutions should give offenders what they deserve by depriving them of liberty rather than by imposing physical pain.^38^ Second, if a kidney nephrectomy is just a way to give offenders what they deserve, the benefits of obtaining a kidney for transplant become weirdly irrelevant to the justification of doing so, and we would lack reasons to make the nephrectomy as painless as possible. This cannot be right.

Another way for the retributivist to justify reducing the sentences of those willing to donate, at least in principle, is to argue that donating an organ improves the moral desert of offenders, such that they come to deserve less punishment. Nevertheless, it is unclear whether this strategy could succeed in justifying the proposal from a retributivist perspective. Admittedly, it is hard to deny that donating an organ for altruistic reasons greatly improves one’s moral desert if such a thing as moral desert exists. However, Bill H.2333 offers sentence reduction as a reward for donating. Presumably, donating in exchange for a sentence reduction increases desert much less, if at all. Of course, some incarcerated might still donate for purely altruistic reasons rather than to obtain a sentence reduction, but how would we know? Thus, the explicit offer of a sentence reduction as a reward for donating undermines the rationale for offering a sentence reduction to incarcerated donors on retributivist, desert-based grounds.

While the above is arguably the “standard” version of retributivism,^39^ alternative fairness-based versions of retributivism are not, as such, concerned with inflicting suffering in proportion to the gravity of the crime but rather with the broader notion of making offenders *repay* the benefits they have unjustly obtained by breaking the law.^40^ Setting aside the question of whether most offenders indeed get any advantage from breaking the law, their organs are surely not such an advantage. Admittedly, the proponent of fairness-based retributivism need not demand that the benefits punishment takes from offenders are of the same *kind* as the benefits the offender unjustly obtained by breaking the law. It *is* possible for punitive repayment to take the form of giving up an organ. However, it would be strange to allow punitive repayment to take this form in a society prohibiting organ commodification.^41^ Thus, it is hard to justify Bill H.2333 according to the prominent version of retributivism surveyed here.

### Communication

According to the *communicative theory* of punishment, the purpose of punishment is to communicate something. The exact details of what punishment is supposed to communicate vary, but a standard version might look something like the following: Punishment expresses authoritative censure^42^ of the punished act as a moral wrong;^43^ constitutes an attempt to persuade the offender to recognize his conduct as wrongful,^44^ and as something he should repent and apologize for, ideally facilitating a reconciliation with the victim and the rest of the community;^45^ and communicates that the community takes the wrong done to the victim seriously, disavows the actions of the offender, and reaffirms the community’s commitment to the violated rights of the victim.^46^ One important aspect of the communicative theory is that it implies that the legal system, including its corrective institutions, should address the incarcerated as responsible moral agents who remain members of the community.^47^

The message sent by offering sentence reductions to incarcerated people willing to donate a kidney or some bone marrow is difficult to reconcile with the aims of the communicative theory for several reasons. First, this offer signals that part of the incarcerated’s sentence is a *mere bargaining chip* that can be traded away in the pursuit of another end (more transplantable organs). This is as antithetical to the use of punishment to express censure as allowing sentence reductions to be purchased outright and reduces the efficacy with which punishment can be used to convey censure. The expression of censure is not a fit “object” for bartering. A legal system that expresses a willingness to reduce the severity with which it censures (i.e., reducing sentences) in exchange for a sufficiently attractive offer thereby indicates that its expression of censure was never really sincere. Second, indicating a willingness to reduce the sentence of offenders who do something completely unrelated to their crime would arguably express that the community does not, in fact, take the wrong done to the victim that seriously. Those who have been severely harmed by a crime can hardly be blamed for feeling that a community that shortens the offender’s sentence because he donated an organ does not take the wrong they have suffered quite as seriously as it should, even if they recognize the value of obtaining an organ that can be used to prolong the life of someone else. Third, offering a sentence reduction in exchange for the donation of a kidney or some bone marrow arguably also fails to treat the incarcerated as members of the community. It signals that the state sees the punishment as something that can be *leveraged* to extract a good, an organ, from the offender rather than something that is used to address the offender in the hope of making him recognize that he has done wrong, repent, apologize, and ultimately reconcile with the community.

One might try to defend Bill H.2333 from these objections by arguing that the donation of a kidney can constitute a form of community service capable of realizing the aims of communicative punishment by causing the offender to make up her wrongdoing to the community in a way that “constitutes an attempt to persuade her to face up to the wrong she has done”^48^ and ideally causes her to recognize her wrong, and see the community service as a sincere apology for her wrongdoing. However, this strategy faces two problems. First, community service best serves the purposes of communicative punishment if the work assigned to the offender clearly relates to her offense.^49^ Presumably, only offenses that damaged the organs of one’s victim would stand in this relation to donating an organ. Second, and more importantly, community service is usually “work” in a way that donating an organ is not. A kidney nephrectomy is a one-time event, whereas community service is extended in time. Given that the purpose of communicative punishment is, *inter alia*, to make the offender recognize that what she did was wrong, community service must arguably be extended in time in a way that a kidney nephrectomy is not to give the offender adequate time to come to this recognition. Thus, Bill H.2333 is difficult to reconcile with the communicative theory of punishment.

It is also worth noting that some of the objections to the proposal raised in the public debate can be seen as drawing on intuitions best captured by the communicative theory. For instance, Kevin Ring, president of Families Against Mandatory Minimums, a Washington, DC-based criminal justice reform advocacy group, told the AP that “[p]romoting organ donation is good. Reducing excessive prison terms is also good. Tying the two together is perverse.”^50^ The perverseness seemingly arises because paired together, these things affect what society expresses about the involved parties.

## Conclusion

Offering to reduce the sentences of those among the incarcerated who become living donors of either organs or bone marrow is a problematic proposal for many reasons. Some such reasons are similar to those critiques often made of so-called organ markets, namely that it may be challenging to ensure proper consent when donors are vulnerable. However, there are also concerns that are more specifically related to the proposal. One aspect pertains to equality of opportunity. The proposal provides the opportunity for a sentence reduction for some but not for others, and the distribution of these opportunities reflects circumstances largely beyond the control of the incarcerated (such as their age and physical condition). Furthermore, the proposal likely conflicts with why we punish in the first place. The proposal is at odds with the non-consequentialist general deterrence defended by Tadros, retributivism, and communicative theories of punishment. Among the theories examined, only the purely consequentialist version of general deterrence might find the practice of granting sentence reductions in exchange for donation palatable. Given the prominence of the theories of punishment we surveyed in this paper, the upshot of the latter observation is that the proposal presupposes the truth of a narrow and controversial subset of the reasons why we punish and sets aside others.
